# Rare variants in *GPR3* in POI patients: a case series with review of literature

**DOI:** 10.1186/s13048-023-01282-3

**Published:** 2023-11-03

**Authors:** Shuting Ren, Feng Zhang, Lingyue Shang, Xi Yang, Yuncheng Pan, Xiaojin Zhang, Yanhua Wu

**Affiliations:** 1 Obstetrics and Gynecology Hospital, NHC Key Lab of Reproduction Regulation (Shanghai Institute for Biomedical and Pharmaceutical Technologies), State Key Laboratory of Genetic, Engineering at School of Life Sciences, Fudan University, Shanghai, 200011 China; 2 Shanghai Key Laboratory of Female Reproductive Endocrine Related Diseases, Shanghai, 200011 China; 3 National Demonstration Center for Experimental Biology Education, School of Life Sciences, Fudan University, Shanghai, 200433 China

**Keywords:** Premature ovarian insufficiency (POI), G protein-coupled receptor 3 (GPR3), Whole-exome sequencing (WES), Single nucleotide variant (SNV)

## Abstract

**Background:**

Premature ovarian insufficiency (POI) is a highly heterogeneous disease, and up to 25% of the cases can be explained by genetic causes. G protein-coupled receptor 3 (GPR3) plays an important role in oocyte arrest, and *Gpr3*-deficient mice exhibited POI-like phenotypes.

**Case presentation:**

We identified two heterozygous missense variants of *GPR3*: NM_005281: c.C973T (p.R325C) and c.G772A (p.A258T) in two sporadic Han Chinese POI cases through whole exome sequencing and genetic analysis. The two patients were diagnosed as POI in their late 20s, presenting elevated serum levels of follicle stimulating hormone and secondary amenorrhea. Both variants are very rare in the population databases of ExAC, gnomAD and PGG.Han. The affected amino acids are conserved across species and the mutated amino acids are predicted deleterious with bioinformatics prediction tools and the protein three-dimensional structure analysis.

**Conclusions:**

It is the first report of rare *GPR3* variants associated with POI women, providing an important piece of evidence for *GPR3* as a candidate gene which should be screened in POI. This finding suggested the necessity of including *GPR3* in etiology study and genetic counseling of POI patients.

**Supplementary Information:**

The online version contains supplementary material available at 10.1186/s13048-023-01282-3.

## Background

Premature ovarian insufficiency (POI) is a common female reproductive disease characterized by menstrual disturbance (amenorrhea or oligomenorrhea) combined with elevated serum follicle stimulating hormone (FSH) level (> 25 IU/L) before 40 years of age [1]. A recent study showed that the prevalence of POI is 3.7% in women [2]. Due to the reduced ovarian function, POI patients suffer from menstrual disorders and fertility difficulties, and they also display higher likelihoods of developing many other long-term physical conditions and mental diseases than normal women.

The causative factors of POI are highly heterogeneous, including genetic factors, autoimmune diseases, iatrogenic injuries and among others. It is estimated that genetic factors account for approximately 25% of POI patients [3]. Especially in recent years, many pathogenic genetic variants including single nucleotide variants (SNVs), small-scale insertions and deletions (Indels) and copy number variations (CNVs) have been characterized in familial or sporadic POI patients with the usage of high-throughput sequencing technology [4–6]. These variants are found to locate in genes mainly involved in meiosis, DNA damage response, steroidogenesis, follicle activation and development, etc. [5].

The human *G protein-coupled receptor 3* (*GPR3*), located on 1p36.11, is predominantly expressed in oocytes, testis and brain [7]. *GPR3* (NM_005281) sencodes a protein of 330 amino acids with seven transmembrane domains, which belongs to the G protein-coupled receptor family and can activate Gs protein and elevate cAMP levels in oocytes [7, 8]. GPR3 has been identified as a crucial factor in the maintenance of meiotic arrest and maturation in oocytes through ex vivo studies [9–11]. Female mice that lack *Gpr3* develop premature ovarian aging due to spontaneous resumption of meiosis in antral follicles, exhibiting reduced fertility in young age and severe infertility in old age [7, 12]. These results altogether suggested *GPR3* as an attractive candidate gene of human POI. Several studies therefore tried to identify disease-associated variants of *GPR3* in POI patients, however all the results were disappointing [13–15]. Whether *GPR3* could be classified as a candidate gene of POI has become a concern. In the present study, our cohort comprises 156 Han Chinese women with sporadic POI as previously described [16, 17]. Two rare missense variants in *GPR3* were identified in two cases.

## Case presentation

### Study participants

One hundred fifty six Chinese women with sporadic POI have been included in our study at the Obstetrics and Gynecology Hospital of Fudan University between February 2017 and March 2022. The informed consent was obtained from all participants. The inclusion criteria consisted of amenorrhea for at least 4 months before 40 years of age and two serum FSH levels greater than 25 IU/L with intervals of more than 4 weeks. Women with ovarian surgery or radiotherapeutic or chemotherapeutic interventions were excluded. Genomic DNA was extracted from peripheral blood samples using the QIAamp DNA Blood Mini Kit (QIAGEN).

### Whole exome sequencing (WES) and data processing

Genomic DNA was then subjected to WES at iGeneTech Bioscience (Beijing, China). Raw data were mapped to the human reference genome sequence (GRCh37/hg19) using the Burrows-Wheeler Alignment tool. The Genome Analysis Toolkit was used to accomplish the variant calling. All variants were further annotated with ANNOVAR software. The processing of genetic analysis was as previously described [17]. Briefly, the medium and high-quality variations were retained, and then the genetic variants in the exonic and splicing regions were chosen. Variant filtering was performed based on a minor allele frequency (MAF) ≤ 1‰ in population databases of the Exome Aggregation Consortium (ExAC), the Genome Aggregation Database (gnomAD) and the Han Chinese Genomes Database (PGG.Han). Synonymous mutations were filtered out. After searching for *GPR3* variants, only two missense variants located in *GPR3* (NM_005281) were identified and then they were investigated by SIFT, MutationTaster, CADD and DANN for further analysis. Finally, all of these two *GPR3* variants carried by F057 and F086 respectively predicted to be deleterious were retained.

### Clinical case report

Clinical information of F057 and F086 are summarized in Table 1. There was no history of any ovarian surgery, uterine surgery, radiotherapy, chemotherapy or autoimmune thyroid disease in the two patients.

**Table 1:** Clinical characteristics of the two POI subjects carrying *GPR3* variants

Characteristic	F057	F086
First menses (years old)	14	14
Age of POI (years old)	27	29
Weight (kg)	44	47.5
Height (cm)	159	162
FSH (IU/L)	68.34	114.63
LH (IU/L)	8.61	50.16
RPL (ng/mL)	7.64	NA
E2 (pg/mL)	< 20	17.08
P (pg/mL)	0.38	0.33
T (ng/mL)	0.37	0.34
AMH (ng/mL)	0.01	NA
Size of ovary (left/right) (mm)	17 × 12 × 8/14 × 13 × 10	14 × 12 × 10/14 × 12 × 11
Size of follicle (left/right) (mm)	Not obvious	Not obvious

*FSH* Follicle-stimulating hormone, *LH* Luteinizing hormone, *PRL* Prolactin, *E2* Estradiol, *P* Progesterone, *T* Testosterone, *AMH* Anti-Müllerian hormone, *NA* Not available

F057 had normal physical development and had spontaneous menarche at the age of 14 with regular cycle. Her menstrual cycle became irregular when she was 26 years old. She was diagnosed with POI one year later and was administered with femoston for maintenance of menstruation. As shown in Table 1, her hormone examination exhibited an increased level of FSH (68.34 IU/L) and a rather low AMH level (0.01 ng/mL). Ultrasound examination results showed she had relatively small ovarian volume, without any obvious follicles on both sides.

F086 reported regular pubertal development with spontaneous menarche at 14 years of age, but the length of her menstrual cycle is about 20 days and she had severe menorrhagia. Her irregular menstruation became even worse after the age of 19 and she was diagnosed with POI at the age of 29. The results of hormone evaluation showed an elevated level of FSH (114.63 IU/L) and a decreased E2 concentration (17.08 pg/mL). Ultrasound examination displayed that she also had small ovaries without any obvious follicles on both sides.

### Identification of *GPR3* variants

**Fig. 1:**
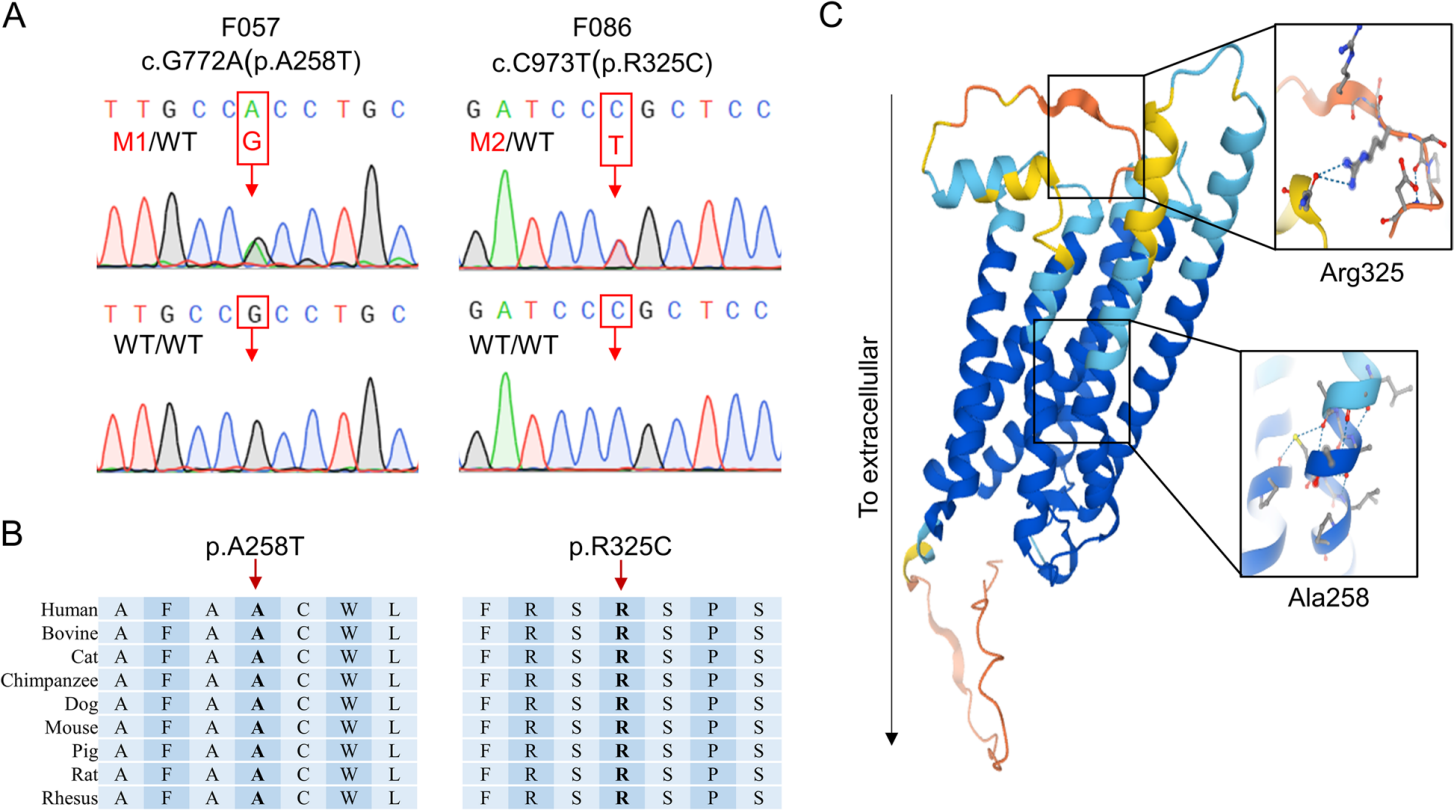
Identification of *GPR3* variants in two POI patients. **A** Sanger sequencing confirmed the heterozygous *GPR3* variants in patients. The red arrows indicate the positions of the two variants. **B** The amino acid residues of GPR3 corresponding to the identified variants are conserved across species. The red arrows indicate the positions of the affected residues. **C** Overview of the predicted structure of GPR3 protein. The enlarged boxes indicate the locations of the affected residues

Two different rare variations in the coding region of *GPR3* were identified in F057 and F086 respectively (Table 2). The authenticity of two variants was first confirmed by Sanger sequencing (Fig. 1A). However, F057’s parental samples were unavailable. Sanger sequencing revealed that F086's variation was inherited from her mother, who had irregular menstruation from the age of 21, followed by sporadic menstruation.

**Table 2:** Overview of the rare *GPR3* variants identified in POI patients

Subject	cDNA change^a^	Protein change	Minor allele frequency^b^			Functional prediction^c^			
			**ExAC**	**gnomAD**	**PGG.Han**	**SIFT**	**MutationTaster**	**CADD**	**DANN**
F057	c.G772A	p.A258T	0.0001	0.0002	0	Deleterious	Disease causing	2.61	0.987
F086	c.C973T	p.R325C	0.0001	0.0002	0	Deleterious	Disease causing	5.121	0.999

^a^The GenBank accession number of *GPR3* is NM_005281

^b^Minor allele frequencies were estimated according to the ExAC (East Asian), gnomAD (East Asian) and PGG.Han databases

^c^Mutation assessment using the SIFT, MutationTaster, CADD and DANN tools. Higher CADD and DANN values indicate that mutations are more likely to have deleterious effects. The CADD threshold is usually set at 4 and 0.93 is used for the DANN cutoff

F057 was found to carry a heterozygous *GPR3* c.G772A (p.A258T) variant and F086 carried a heterozygous *GPR3* c.C973T (p.R325C) variant. Both variants have low frequencies in the ExAC and gnomAD population databases and are absent in the PGG.Han database, which includes genomic data of 114,783 Han Chinese individuals [18]. The c.G772A (p.A258T) variant was predicted to be deleterious by SIFT, MutationTaster and DANN, while the c.C973T (p.R325C) variant was harmful according to all mutation assessments (Table 2).

Additionally, both variants are highly evolutionarily conserved across species (Fig. 1B). According to the three-dimensional structure of wild-type GPR3 protein obtained from the Uniprot database (Uniprot entry: P46089), it is found that Ala258 was located in the sixth transmembrane domain (Fig. 1C) and the p.A258T variant may affect the protein structure. Additionally, the Arg325 was located in intracellular surface and the change from arginine to cysteine could affect the interaction with Ser324, which may lead to dysregulation of intracellular cAMP signaling. In addition, according to 3D structure prediction, Arg325 also interact with Glu302 at the end of the seventh transmembrane domain, thus p.R325C variant may also affect this interaction and the whole protein structure.

### Literature review

We searched the PubMed database using text words relating to “*GPR3*” and “POI” or “oocyte” and summarized the representative supporting evidence for *GPR3* as a candidate gene for POI (Table 3). Previous clinical studies of *GRP3* variants in POI patients have also been reviewed.

**Table 3:** Representative supporting evidence for *GPR3* as a POI candidate gene

Index	Research type	Research object	Research method	Phenotype/results	Ref
1	Animal model	*Gpr3*^−/−^ mice; C57BL/6J	Knock out	Resumption of meiosis in most of the oocytes within antral follicles	[7]
2	Animal model	*Gpr3*^−/−^ mice; C57BL/6J and CD1	Knock out	Accelerated age-dependent reduction of fertility; premature resumption of meiosis I and impaired meiotic competence; increased FSH levels and shorter estrous cycles in aging females	[12]
3	Animal model	*Gpr3*^−/−^ mice; C57BL/6J	Knock out	Lower amounts of CDK1 and decrease of meiotic competence in oocytes	[19]
4	Ex vivo cell experiment	Porcine oocytes	Knock down; overexpression	Abnormal meiosis resumption; changes in cyclin B, cAMP and cGMP levels	[11]
5	Clinical report	82 Caucasian POF women	Polymerase chain reaction (PCR) and denaturing high-performance liquid chromatography (DHPLC)	No disease-associated variants in *GPR3*	[13]
6	Clinical report	100 Chinese POF women	PCR and sequencing	No disease-associated variants in *GPR3*	[14]
7	Clinical report	269 POI women (mostly Caucasian)	Candidate genes sequencing	No disease-associated variants in *GPR3*	[15]
8	Clinical report	156 Chinese POI women	WES	Two rare missense variants in *GPR3* identified in two patients	This paper

GPR3 was first characterized as a pivotal factor for oocyte meiosis in 2004, when Mehlmann et al*.* found that GPR3 maintained the prophase I arrest in mice [7]. Most oocytes from *Gpr3* knockout mouse resumed meiosis within antral follicles spontaneously. Ledent et al*.* found that *Gpr3* deficient mice were subfertile and further demonstrated that Gpr3 protected and possibly rescued oocytes from aging [12]. He also found increased oocyte fragmentation in superovulated *Gpr3*^*−/−*^ female mice, suggesting a higher proportion of atresia and poor meiotic oocytes. In addition, *Gpr3* knockout mice were found to have POI-like phenotypes including increased FSH levels and shorter estrus cycles [12]. Phenotypes in *Gpr3* knockout mice were also mimicked by specific reduction of *Gpr3* in the mouse oocyte using RNAi techniques [9]. Oocytes injected with *Gpr3* siRNA lost their ability to maintain meiotic arrest and the GVBD rate observed was very close to the rate seen in *Gpr3* global knockout mice. Similar experimental results were obtained in porcine oocytes [11]. Injection of siRNA targeting *GPR3* stimulated meiotic resumption of oocytes, while overexpression of *GPR3* inhibited meiotic maturation of porcine oocytes. Mechanism study further found that *Gpr3* was expressed and remained active before cAMP is required to maintain meiotic arrest, indicating another role of Gpr3 in the acquisition of oocyte meiotic competence [19]. Taken together, these functional studies and animal models all suggested *GPR3* as an attractive candidate gene for human POI.

However, when Ertug Kovanci et al.first tried to find any sequence variants in 82 American premature ovarian failure (POF) women, he failed to detect any potentially disease-associated *GPR3* variants among them [13]. Similarly, no *GPR3* mutations affecting the coding sequence was found in 100 Chinese POF patients in 2010 [14]. In a recent study, 269 POI patients were screened for several causative/candidate POI genes including *GPR3* by Ion Torrent semiconductor sequencing and none of them presented any pathogenic variants of *GPR3* [15]. Therefore, our study is the first report to identify rare and likely pathogenic variants of *GPR3* in two Chinese POI patients, presenting evidence that *GPR3* is involved in POI pathogenesis.

## Discussion and conclusions

Identifying causative variants in POI patients has been challenging with the high heterogeneity in etiology. Although 159 POI-related genes have been listed in the Ovarian Kaleidoscope database (OKdb), the genetic causes remain to be elucidated in the majority of clinical POI patients [5]. Herein, we identified two rare *GPR3* variants in a cohort of 156 sporadic POI patients, and the harmfulness of them were further confirmed by bioinformatics software. To exclude the potential contribution of other pathogenic variants in F057 and F086, we performed the genetic analysis on all the variants from the two patients. As shown in the supplementary Table 1, after a series of filtering steps, there was no rare and deleterious variant of any other POI causative/candidate genes except *GPR3* in both patients. However, considering that the genetic etiological spectrum of POI is still expanding, it is undeniable that there is the possibility that other novel POI causative/candidate genes might involve in the pathogenesis of POI.

POI is a complex disorder with high genetic heterogeneity. No POI-related gene is implicated in more than 5% of sporadic cases, and most POI genes could not be replicated in different reports. For example, *FMR1* premutation is one of the most common causes of POI in western countries. Around 11–14% of familial and 2–6% of sporadic POI cases are associated with *FMR1* premutation [20]. However, there is a rather lower contribution (< 1%) of *FMR1* premutation in Chinese POI women [21–23]. More recently, a large cohort of 375 POI patients have been subjected to genetic analysis by targeted sequencing (88 POI-related genes) and whole exome sequencing [24]. Pathogenic variants in 50 POI genes were detected, most of which were identified in only one patient. More importantly, no variant was identified in the other POI genes at all. Taken all these together, we think that ethnicity difference, limited sample size and genetic heterogeneity of POI may also affect the contribution of *GPR3* variants in different POI studies.

*GPR3* is predominantly expressed in oocytes [7]. Studies with genetic or oligonucleotide-mediated ablation of *GPR3* in mice provided supporting evidence that GPR3 is required to maintain high cAMP levels in the oocytes and meiotic arrest [7, 9]. *GPR3* is also expressed in Xenopus, porcine and human oocytes, suggesting a conserved function across species [10, 11, 25]. A phenotype reminiscent of human premature ovarian failure was also reported in *GPR3* deficient mice by Ledent et al. [12]. Resumption of meiosis in *Gpr3* siRNA-injected oocytes was dose-dependent [9], suggesting that the amount of Gpr3 is important. It also suggests a possibility for the pathogenicity of heterozygous deleterious variants of *GPR3*. However, the molecular mechanism of GPR3 affecting meiosis is still unclear, whether there are undiscovered protein mediators in the related pathway needs further study. Moreover, the pathogenesis of *GPR3* variation identified in this work remained to be further elucidated in future by molecular methods or animal models.

In conclusion, we reported for the first time two rare variants of *GPR3* associated with POI patients, providing important evidence for *GPR3* as a candidate gene that should be screened for in POI patients. Several previous reports have not found any *GPR3* variation in POI patients, probably due to the high heterogeneity of POI in etiology. A larger cohort of POI patients is expected to precisely assess the contribution of *GPR3* variation in POI and to define the genotype–phenotype correlations in women carrying *GPR3* variants.

### Supplementary Information

**Additional file 1: Supplementary table 1.** Filtering steps of two WES data processing. **Supplementary table 2.** Primers used for Sanger sequencing. **Supplementary table 3.** List of 101 known POI causative genes. **Supplementary table 4.** List of 92 POI candidate genes.

## Data Availability

The datasets used and/or analyzed during the current study are available from the corresponding author on reasonable request.

## Abbreviations

AMH: Anti-Müllerian hormone
CNV: Copy number variation
DHPLC: Denaturing high-performance liquid chromatography
E2: Estradiol
ExAC: Exome Aggregation Consortium
FSH: Follicle stimulating hormone
gnomAD: Genome Aggregation Database
GPR3: G protein-coupled receptor 3
LH: Luteinizing hormone
MAF: Minor allele frequency
OKdb: Ovarian Kaleidoscope database
P: Progesterone
PCR: Polymerase chain reaction
PGG.Han: Han Chinese Genomes Database
POF: Premature ovarian failure
POI: Premature ovarian insufficiency
PRL: Prolactin
SNV: Single nucleotide variant
T: Testosterone
WES: Whole-exome sequencing
