# Supplementary material for: Rare variants in GPR3 in POI patients: a case series with review of literature
Source: J Ovarian Res. 2023 Nov 3;16:210. doi: 10.1186/s13048-023-01282-3 (PMC10623876; doi:10.1186/s13048-023-01282-3)
Supplement: Supplementary file 1 — Additional file 1: Supplementary table 1. Filtering steps of two WES data processing. Supplementary table 2. Primers used for Sanger sequencing. Supplementary table 3. List of 101 known POI causative genes. Supplementary table 4. List of 92 POI candidate genes. [file 13048_2023_1282_MOESM1_ESM.zip › Ren_GPR3_supplementary_with_changes_marked.docx]

| **step** | **Number of variants** | |
| --- | --- | --- |
|  | **F057** | **F086** |
| All data called by WES | 142950 | 147591 |
| High and medium calling quality | 87894 | 93908 |
| In exonic and splicing regions | 19891 | 20906 |
| Allele frequencies≤ 0.001 ^a^ | 325 | 360 |
| Filter out synonymous SNVs | 220 | 248 |
| Nonsense, frameshift, non-frameshift Indel, splicing site, or deleterious missense variants ^b^ | 133 | 153 |
| Known causative/candidate POI genes ^c^ | 1 | 1 |

**Supplementary table 1** Filtering steps of two WES data processing

^a^ Allele frequencies were estimated according to 1KG Project, ExAC, and gnomAD databases

^b^ All missense variants were assessed using the SIFT, PolyPhen-2, MutationTaster, and CADD tools. Missense variants predicted to be deleterious by at least two software were retained

^c^ POI genes were obtained from previous work in the laboratory and shown in supplemental Tables 3 and 4

**Supplementary table 2** Primers used for Sanger sequencing

| Primer name | Primer sequences (5’ to 3’) |
| --- | --- |
| *GPR3*-F | CACATGTGGCGTGGTTTATCC |
| *GPR3*-R | CATTCTGGAACCGTGGAGCC |
